# The effects of temperature and dispersal on species diversity in natural microbial metacommunities

**DOI:** 10.1038/s41598-019-54866-9

**Published:** 2019-12-04

**Authors:** Elodie C. Parain, Sarah M. Gray, Louis-Félix Bersier

**Affiliations:** 1 University of Fribourg, Department of Biology - Ecology and Evolution, Chemin du Musée 10, 1700 Fribourg, Switzerland; 2 Yale University, Department of Ecology and Evolutionary Biology, 165 Prospect Street, New Haven, CT 06520 USA

**Keywords:** Climate-change ecology, Community ecology

## Abstract

Dispersal is key for maintaining biodiversity at local- and regional scales in metacommunities. However, little is known about the combined effects of dispersal and climate change on biodiversity. Theory predicts that alpha-diversity is maximized at intermediate dispersal rates, resulting in a hump-shaped diversity-dispersal relationship. This relationship is predicted to flatten when competition increases. We anticipate that this same flattening will occur with increased temperature because, in the rising part of the temperature performance curve, interspecific competition is predicted to increase. We explored this question using aquatic communities of *Sarracenia purpurea* from early- and late-successional stages, in which we simulated four levels of dispersal and four temperature scenarios. With increased dispersal, the hump shape was observed consistently in late successional communities, but only in higher temperature treatments in early succession. Increased temperature did not flatten the hump-shape relationship, but decreased the level of alpha- and gamma-diversity. Interestingly, higher temperatures negatively impacted small-bodied species. These metacommunity-level extinctions likely relaxed interspecific competition, which could explain the absence of flattening of the diversity-dispersal relationship. Our findings suggest that climate change will cause extinctions both at local- and global- scales and emphasize the importance of intermediate levels of dispersal as an insurance for local diversity.

## Introduction

The species diversity of an ecosystem plays a major role in determining the health of the environment and the amount of ecosystem services that it can provide^1–3^. Biodiversity, however, is highly threatened by the various environmental changes induced by human activities^4^. It is essential to identify the processes that are important for maintaining biodiversity and how these processes will be affected by environmental change. One of the major anthropogenic impacts is the fragmentation of the landscape, leading to suitable habitat patches being isolated in a matrix of unfavorable environment^5,6^. The ability of a species to disperse between communities has been shown to play an important role for sustaining a high level of species diversity at both the local and regional scale^7–10^. Immigration thus maintains diversity by creating an influx of rare and less competitive species. Without this influx, the best competitor in a local community is likely to dominate the system and ultimately reduce species diversity^11^.

The model of Mouquet and Loreau^7^ predicts that an intermediate level of dispersal is optimal for maximizing diversity at the local scale in these competition-driven metacommunities. According to this theory, when no dispersal between communities occurs, alpha-diversity is expected to be low because competitively-dominant species are likely to drive other species to local extinction. At the other extreme, with high dispersal frequency, alpha- and gamma-diversity will also be low because species composition is homogenized at all scales, making the system behave as in a single patch.

This diversity-dispersal relationship has been investigated for fragmented patches under stable conditions^7,12^. However, little is known about if an intermediate level of dispersal will still maintain the highest level of diversity when the metacommunity is subject to environmental change. Global warming, in particular, is expected to strongly impact the environment and species persistence^13,14^ and Thompson *et al*.^15^ showed that this hump-shaped relationship may not be maintained with an increase in temperature. Temperature increase affects species interactions by increasing attack rate; in the rising part of the temperature performance curve, the Arrhenius equation has been suggested to adequately capture this relationship in poikilothermal species^16,17^. Metabolic rates increase with temperature, which will cause consumers to have higher per capita attack rates on their resources. This temperature-induced increase in attack rates will cause greater interspecific competition due to an increase in niche overlap, irrespective of other demographic parameters^18,19^. The effect of temperature on the change in “standardized effective interaction” is derived in Parain *et al*.^20^, equation 13, and shows that interspecific interactions are expected to increase disproportionately with temperature compared to intraspecific interactions. As proposed by Mouquet and Loreau^7^ and extended by Kneitel and Miller^8^, stronger interspecific competition is one mechanism that will flatten the hump-shaped relationship between diversity and dispersal: less competitive species will become locally extinct^21^ and alpha-diversity will decrease if the system contains the original gamma-diversity and if the dispersal rate is constant between patches. As a consequence, an increase in competition induced by warming may hinder the positive effect of intermediate dispersal for biodiversity maintenance.

Because the strength of competitive interactions influences the shape of the diversity-dispersal relationship, the successional stage of competition-driven communities is also expected to affect this relationship. Early-successional communities are typically composed of pioneer species, characterized by high growth rates, colonization abilities, and tolerant to disturbances^22,23^. The colonization of new habitat patches is usually a stochastic event that can lead to the presence of several species occurring in similar niches, which is expected to generate high niche overlap and thus strong average interspecific competition in the communities^24^. At this stage, the competition-colonization trade-off is also expected to generate labile community structures^25^. The dynamics of these communities are moreover difficult to predict as they are additionally governed by processes such as priority effects and species sorting^26^. In older communities, the species present are likely to be those that have already gone through the competition filter^27^. Therefore, late-successional communities should be composed of stronger competitors and have lower average niche overlap than early succession communities^23^. However, these superior competitors face a trade-off between competitive ability and resistance to perturbations^28,29^. Since early- and late-successional communities have different organization and dynamics, it is expected that they will respond differently to the interacting effects of global warming and dispersal rates.

In this study, we aim to understand the combined effects of temperature change and of varying dispersal rates on the diversity in metacommunities from early- and late- successional stages. Specifically, we would like to test how temperature affects the hump-shaped relationship of the diversity-dispersal relationship, and if successional stage of a community affects this response. In order to answer our question experimentally, we used the inquiline communities of a carnivorous plant, *Sarracenia purpurea*. This system is appropriate to test questions pertaining to metacommunities^30^ because each leaf acts as a natural, bounded habitat, holding communities that are linked to one another through dispersal. Microorganisms disperse passively in drops of water splashed to neighboring leaves during rain events, or carried by insects moving from one leaf to another^31^. This type of dispersal can easily be simulated in experiments by indiscriminately pipetting microorganisms between communities. This is the approach chosen by Kneitel and Miller^8^, who used this system in its native range to test for the unimodal relationship between dispersal and diversity in the presence and absence of the top predator, the larvae of *Wyeomyia smithii*. They found this hump shape only when the predator was absent. In Europe, these communities are generally composed of two trophic levels only, with protists and rotifers forming the top trophic level, preying on bacteria (bottom trophic level). We used this European system in our experiment because it lacks the third trophic level, making it likely that competition, and not predation, is the key factor dictating the dynamics of the horizontal communities formed by the protists.

In our study, we followed experimental early- and late- protist metacommunities during seven weeks. We measured response variables at the local-community level: alpha-diversity, total density, evenness, and community composition (including body size). At the metacommunity level, we recorded beta- and gamma-diversity. Following Mouquet and Loreau^7^, we predict that maximum alpha-diversity will be achieved at intermediate rates of dispersal. This hump-shaped relationship is expected to flatten with increased temperature due to stronger interspecific competition^8^ induced by higher attack rates^20^. The hump-shaped relationship should be most evident in low temperature conditions and in late-successional communities, since interspecific competition is expected to be lowest in these cases. As temperature rises, we predict a flattening of the hump-shaped relationship in both types of communities; for early-successional communities, the combination of high intrinsic niche overlap and of increased competition due to temperature should rapidly disrupt the hump-shaped relationship. The theory of Mouquet and Loreau^7^ also makes predictions for beta- and gamma-diversity, which are both expected to decrease sharply with dispersal. The additional effect of temperature is more difficult to predict, but we can expect gamma-diversity to decrease because higher interspecific interactions with high temperature should lead to more extinctions. For this reason, we also investigated evenness, which is known to correlate with species survival^32^. Finally, we predict that, in accordance with the temperature-size rule^33^, higher temperatures would favor smaller-bodied species, thus shifting the species composition to predominately small-bodied species in this treatment. We do not expect the dispersal treatment to change this pattern since high temperature would shift the body size to small species in all patches within the metacommunity.

## Materials and Methods

### Inquiline community field collection

We sampled *S. purpurea* inquiline communities from the site ‘Champ Buet’ in Switzerland, which is situated at 500 m above sea level (CB, 46°36′50″N, 6°34′50″E). Eighty leaves that were nearly open were marked at the beginning of June 2014, and 80 additional nearly opened leaves were marked two weeks later. Thus, the inquiline communities within the leaves were allowed to develop for four weeks (‘late-succession’) and two weeks (‘early-succession’), respectively. This time period was chosen based on the growing season at this field site, in which new leaves are mainly only produced in June and July, on the fast dynamics of the species within the inquiline communities, and because most of the successional processes have been shown to occur early in the life cycle of the leaf ^33–35^. At the end of the four weeks, all 160 leaves were sampled at the same time. The sampled water from each successional stage was place into two separate sterilized 1 L Nalgene bottles. The bottles were brought back to the laboratory and chilled at 4 °C to temporarily slow community dynamics until the set-up of the experiment the following day.

### Experimental set-up

The overall density of the protists was measured for the pooled early- and pooled late- succession communities. The following procedure was then applied to both stages. The water was diluted in order to reach a density of 10’000 individuals of protists per mL and eighty 50 mL macrocentrifuge tubes were filled with a 10 mL aliquot of these dilutions. As a basal food resource, we added 500 µL of an autoclaved Tetramin fish food solution (concentration of 2 mg of solid fish food in 1 mL of DI water) into each tube [following protocols used for the *S. purpurea* system by terHorst^36^ and modified by Parain *et al*.^37^]. This resource is consumed by the bacteria in the system, which are then consumed by the protists and rotifers.

#### Experimental design

The 4 × 4 × 2 factorial design included four dispersal levels (No-, Low-, Medium-, and High-dispersal) and four temperature treatments (Local, −2.5 °C below the local average temperature, +2.5 °C and +5 °C above the local average temperature) and two levels of community succession (Early succession and Late succession). Our temperature treatments were based on the natural June temperatures of the field site (minimum: 10 °C, average: 15.5 °C, maximum: 20.9 °C) according to 30 years of data acquired by WorldClim (www.worldclim.org, accessed January 2017). Each treatment was composed of five tubes forming a metacommunity, totaling 160 tubes, which were placed in Panasonic MIR-154 incubators for the experiment, equipped with new lightbulbs and with light and temperature data loggers to exclude possible unwanted variability in our experiment (See Electronic Supplementary Material, Fig. S1 for details on temperature treatments); all tubes were placed in a randomized design within each incubator and this design was changed after every dispersal event. Dispersal was manipulated twice a week by transferring different numbers of individuals between the tubes of a treatment, according to a protocol adapted from Kneitel and Miller^8^. These dispersal events were done separately for every treatment: the individuals of a treatment were only allowed to disperse within their specific metacommunity. Within each treatment, an aliquot of 100 µl was removed from each of the five tubes and combined into a 15 mL sterile macrocentrifuge tube. This mixture was then diluted with autoclaved DI water according to the dispersal level of that treatment. This dilution was necessary to maintain the same volume of water across all treatments, while allowing different numbers of individuals to disperse according to treatment. For the ‘high dispersal’ treatment, 100 µL of this mixture was returned into each of the 5 tubes without dilution. For the ‘medium dispersal’ treatment, the mixture was diluted ten times and added to each of the 5 tubes, and 100 times for the ‘low dispersal’ treatment. For the no-dispersal treatment, 100 µl aliquots were also removed and re-pipetted into the same tube. We checked the efficiency of our dispersal treatment by analyzing the relationship between alpha- and gamma-diversity, which is expected to approach a 1:1 relationship with higher dispersal^11^. We found that this relationship was indeed the case for our chosen dispersal levels (See Electronic Supplementary Material, Fig. S2).

Following the protocol of Kneitel and Miller^8^, the experiment lasted for 7 weeks, after an initial incubation of 8 days in the “Local Temperature” incubator. Communities were fed once a week with 500 µL of fish food at the same concentration as described above. Every week, we sampled 100 µL of water in each tube after gently mixing, and estimated the density and composition of protist- and rotifer- species. Individuals were identified according to their morphology and categorized into 18 morphospecies and three size classes (small, medium, and large; see Electronic Supplementary Material, Table S1). We used an inverted microscope at 100x magnification and a Thoma cell microscope plate to count the protists and rotifers. The references Lee, Leedale and Bradbury^38^ and Streble and Krauter^39^ were used to assist in morphological identification. Presence-absence was used to measure species diversity and it was determined with the entire 100 µL sample; densities of common species were estimated on two grids of the Thoma cell (number of individuals per 0.2 µL). Densities of rare species (observed only outside of the grid) were set to 0.1. Note that due to the large size of the experiment, feasibility, and to the lack of reliable fine-scale molecular information for protists^40^, we chose not to use protist sequence data for species identification; also, our morphology-based approach is logical as we are more interested in functional differences between organisms than on their exact taxonomical status. Note also that we did not assess the dynamics of the bacteria; by regularly adding Tetramin resource, we assumed that the bacteria never represented a limiting factor for protist density in any of the temperature treatments.

### Statistical analyses

We tested the effects of succession, temperature, and dispersal rate on community metrics estimated either at the local-community level (i.e., in each experimental tube) or at the metacommunity level (i.e., in the five tubes of each treatment). For the former, we measured alpha-diversity (number of morphospecies), evenness (Pielou’s index), and total density (total number of individuals per 0.2 µL); for the latter, we measured beta-diversity (Whittaker’s species turnover index measured for the five tubes; see Magurran^41^ pp. 169), and gamma-diversity (total number of morphospecies of the five tubes). We used linear mixed-effects models for metrics measured at the tube level, and generalized least squares models otherwise [functions lme and gls, package nlme^42^]. Mixed effects models were necessary for community metrics measured at the tube level because they were linked by the dispersal treatment. The random factor was tube identity nested in treatment. We considered only random intercept models. Repeated measures were included in all models by using an auto-regression of order 1 (corAR1 argument of the lme and gls function; see^43^). This approach allowed us to estimate the correlation between sequential measurements and to correct p-values accordingly. Note that the replication in our experiment comes from the repeated measures as we have one metacommunity per treatment.

The explanatory variables are succession (early and late) and the two experimental factors (temperature and dispersal). Levels of dispersal rate were coded as 0 (no dispersal), and 1, 2, or 3 for low, medium, and high dispersal, respectively. Temperature was coded as −1, 0, 1, or 2 for the levels −2.5 °C, local condition, +2.5 °C, and +5 °C, respectively. We assessed only the linear effect of temperature, and the linear and quadratic effects of dispersal; the quadratic term was necessary to capture the hump shape of the diversity-dispersal relationship; if the quadratic term was significant, to ascertain the presence of a maximum at intermediate dispersal, we also checked that the difference between the pooled no- and high-dispersal vs. low- and medium-dispersal was significant.

We observed a strong decline in alpha-diversity and evenness during the three first counting sessions followed by a stabilization during the following weeks (see Electronic Supplementary Material, Fig. S3). Consequently, we included sampling session (counted in weeks) as a covariate in the corresponding models, in which we applied a reciprocal transformation to sampling week. For total density and gamma-diversity, only the linear effect of sampling week was included; for beta-diversity, we added a quadratic term as this response variable showed a unimodal pattern with time. For the three diversity measures and evenness, total density (log-transformed) was included as a covariate to account for its possible confounding effect on these metrics.

In all cases, we performed model selection based on AIC according to Zuur (pp. 127)^43^, starting with all interactions between the explanatory variables (succession, temperature, and the linear and quadratic terms of dispersal); we did not consider interactions with the covariates. Residuals of all models were evaluated through visual inspection of QQ-plots and by Shapiro goodness-of-fit tests. The response variables ‘evenness’ and ‘total density’ were log-transformed to reach normality assumptions of the statistical models. Since the effect of successional stage was always strong, for simplicity we report most results separately for early and late succession.

Because the dynamics of the morphospecies may differ according to their body size, we analyzed the proportions of species extinctions according to the size category of the morphospecies with a binomial mixed effect model using taxonomic category as a random effect. We further analyzed how the proportions of ‘small’ vs. ‘medium plus large’ morphospecies changed through time in the early- and late-successional stages (we pooled medium and large species as they yielded qualitatively similar results). We used a binomial mixed-effects model with tube identity as a random factor to account for the repeated measures. Sampling week and the interaction between sampling week and 1) dispersal and 2) temperature were the explanatory variables.

Multivariate analyses evaluated the effects of succession, temperature, dispersal, and sampling week on community composition. We added the pairwise interactions between these four explanatory variables into our model. We computed the dissimilarities between communities in the 160 tubes over the seven weeks, using Jaccard index on presence-absence data [function betadiver of the vegan package, following Koleff *et al*.^44^]. We applied a multivariate ANOVA on the distance matrix with the function adonis in the package vegan in r^45^. We performed 9999 simulations to evaluate the p-values, with permutations constrained within treatments. This does not fully account for the structure of the experiment and the p-values must be considered with caution. However, we were more interested in the contributions, given by the R^2^ values, of the different explanatory variables to the total variability of the community composition than on their statistical significance. To visualize the results, we performed a Correspondence Analysis (CA, function cca of the vegan package) on the data.

## Results

**Figure 1:**
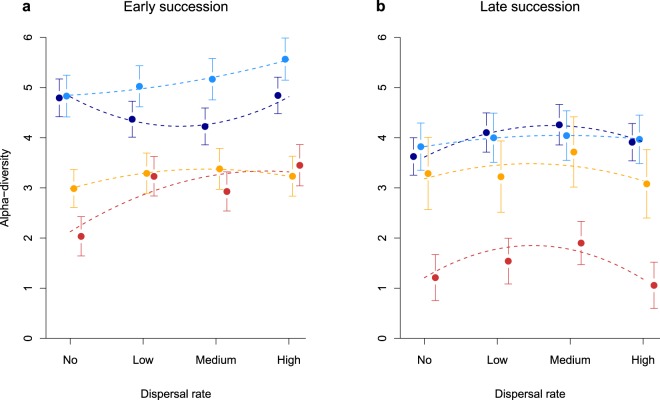
Diversity-dispersal relationship and response of alpha-diversity to temperature increase for communities from (**a**) early- and (**b**) late-successional stages. The temperature treatments are represented as Local Temperature (light blue), Local Temperature −2.5 °C (dark blue), Local Temperature +2.5 °C (orange), Local Temperature +5 °C (red). (**a)** Alpha-diversity in early-successional communities showed a hump-shaped relationship, but only in the high temperature treatments. (**b**) In late-successional communities, the hump shape for alpha-diversity was observed consistently in all temperature treatments. Contrary to our expectation, the observed hump-shaped relationship did not flatten with increased temperatures. Error bars indicate one standard error, and the dashed lines are results of quadratic regressions.

As expected, alpha-diversity in the two successional stages showed different responses. Only temperature had a consistent and negative effect in both stages, while the effects of increasing dispersal rates were different: the relationships were variable in early succession, while the hump shape was observed in all four temperature treatments in late succession (Fig. 1). Table S2 in the Electronic Supplementary Material provides the global results of a linear mixed-effects model for alpha-diversity that includes successional stage. These results show that alpha-diversity was generally lower in late succession, and they highlight the complex interactions between succession, temperature and the linear and quadratic terms of dispersal. Since the effect of succession was strong in all our models, for simplicity we report the following results separately for early- and late-successional stages (Table 1). Here, the variability of the effect of dispersal in the four temperature treatments for the early succession was evidenced by the significant interaction terms between these variables. In accordance with Fig. 1b, these interactions are absent from the best model for late succession. In this stage, the hump shape was consistently observed (the quadratic term was highly significant, and alpha-diversity in low- and medium-dispersal categories was significantly higher than in no- and high-dispersal categories: difference = 0.34, s.e. = 0.12, p = 0.005). However, contrary to expectation, this unimodal relationship between diversity and dispersal did not flatten with increased temperature.

**Table 1:** Alpha-diversity as a function of dispersal- and temperature- treatments in early- and late-successional stages.

Successional stage	Parameters	Estimates	SE	t-value	p-value
early	intercept	4.08	0.23	17.96	<0.001
	temperature	−0.41	0.11	−3.92	0.004
	dispersal	0.53	0.47	1.12	0.29
	(dispersal)^2^	−0.52	0.41	−1.26	0.24
	temp: disp	0.53	0.17	3.11	0.013
	temp: (disp)^2^	−0.14	0.054	−2.54	0.032
	disp: (disp)^2^	0.13	0.090	1.46	0.18
	(week)^−1^	3.54	0.20	17.95	<0.001
	log(density)	−0.27	0.058	−4.65	<0.001
late	intercept	3.17	0.13	23.84	<0.001
	temperature	−0.20	0.050	−3.97	0.002
	dispersal	0.54	0.18	3.07	0.010
	(dispersal)^2^	−0.17	0.056	−3.00	0.012
	(week)^−1^	2.90	0.18	15.69	<0.001

Results of the best linear mixed-effects models with AR1 correlation structure, starting with models that consider all interactions between temperature and the linear and quadratic terms of dispersal. Week of sampling (reciprocally transformed: (week)^−1^) and total density (log-transformed) were included as covariate.

The responses of evenness, total density, and gamma- and beta-diversity also depended on successional stage, and are reported separately for both stages (Electronic Supplementary Material, Tables S3 and S4, Fig. S4). Evenness decreased with temperature in both successional stages. In late-successional stages, evenness obtained a hump shape. Total density was not influenced by any treatment in early-successional stages, but increased with temperature in late succession. Here, there was evidence of a hump-shaped relationship that disappeared with increasing temperature (the interactions between temperature and dispersal were included in the best model, but these terms were not significant). Gamma-diversity was weakly affected by temperature and dispersal in both successional stages. The effect of temperature was consistently negative, which was not the case for dispersal. Beta-diversity was not influenced by our experimental treatments in early succession. It showed a trend similar to gamma-diversity in late succession, with dispersal having a positive effect in lower temperatures and a negative one in higher temperatures. Note that neither gamma- nor beta-diversity markedly decreased with dispersal, as predicted in the theory of Mouquet and Loreau^7^.

**Figure 2:**
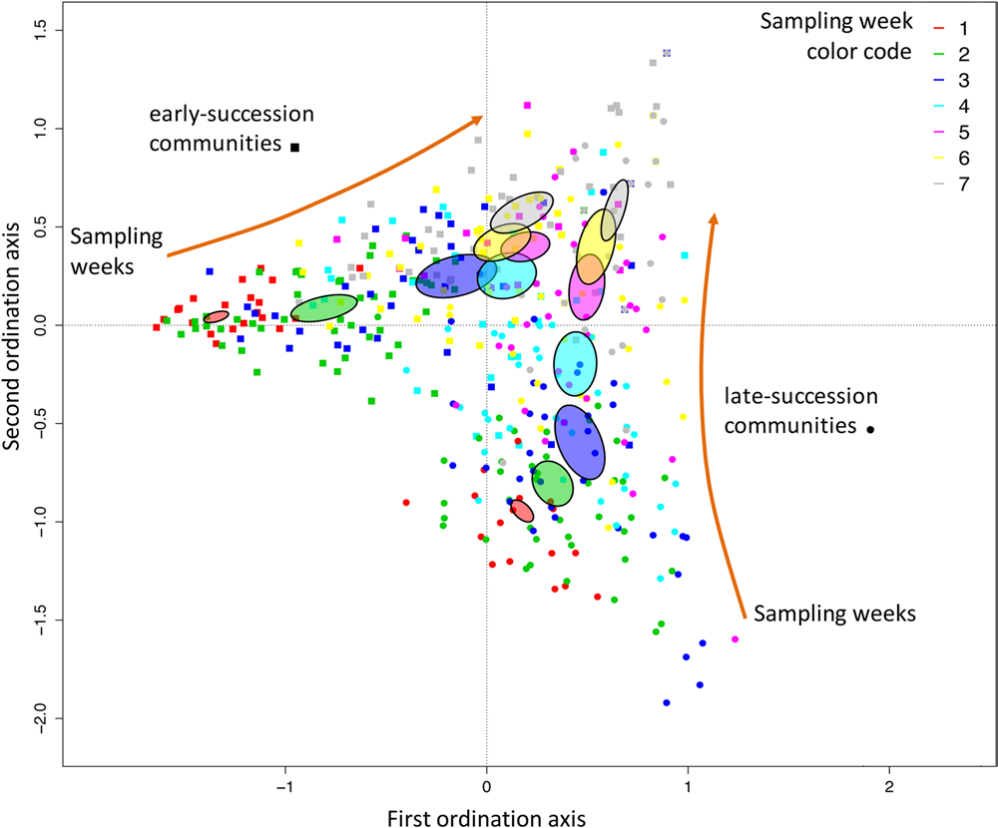
Correspondence Analysis (CA) plot depicting the change in community composition through time (different colors for the sampling sessions 1 to 7), and for early- (square) and late- (circle) successional stages. Distance between points is proportional to compositional dissimilarity. Ellipses are drawn for each sampling session and for early- and late-successional communities; they are centered on the centroid and their size is proportional to the standard error of the coordinates of the corresponding points. Note that the size of the ellipses is larger for the intermediate sampling weeks, indicating higher compositional variability; this is in accordance with the significant quadratic effect of sampling week for beta diversity in the Supporting Information Table S4. Both early and late communities converged toward similar composition, shown by the arrows. Note that the effect of temperature and of dispersal is not shown as their contribution to community composition is minor compared to that of successional stage and sampling week.

Most community metrics were affected by the auxiliary variables “sampling week” (see Electronic Supplementary Material, Fig. S3) and “total density”. Interestingly, the relationship between beta-diversity and sampling week was strongly unimodal in both successional stages (Table S4). This pattern is apparent in Fig. 2, where compositional variability is highest in the intermediate sampling weeks.

**Figure 3:**
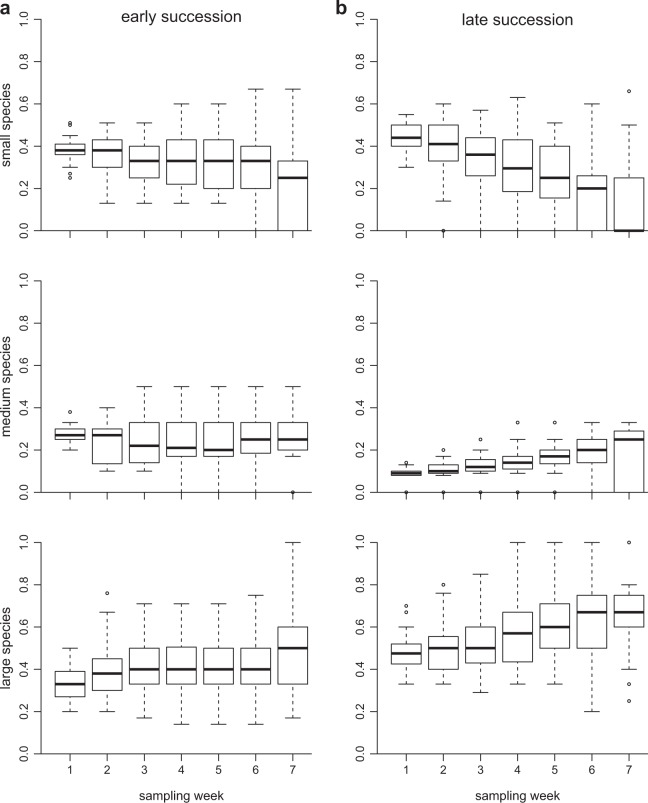
Change through time (sampling week) of the relative number of species in the three categories of body size in the (**a**) early- and (**b**) late-successional stages. Number of species is expressed as percentages within each tube. Note the strong decrease in small species in the late-successional stage.

The size of the morphospecies greatly determined their response to the various treatments. Small-bodied morphospecies were the losers in the experiment; they experienced more extinctions (Electronic Supplementary Material, Table S5 and Fig. S5) and their presence in the community decreased markedly with sampling week (Fig. 3). This trend was more acute with higher dispersal and with higher temperature, especially in the late-successional stage (Electronic Supplementary Material, Table S6 and Figs. S6 and S7), and is contrary to expectation from the temperature-size rule^33^. In accordance with the results of Table 1, evidence of a hump-shape is found for dispersal in the late-successional communities only (Electronic Supplementary Material, Fig. S7b).

The multivariate analysis on species composition revealed the major influence of succession, sampling week, and of their interaction, compared to that of temperature and dispersal (Electronic Supplementary Material, Table S7). Species composition in early- and late-successional communities differed greatly at the start of the experiment, but converged in similarity with time (Fig. 2). The effects of temperature and dispersal were highly significant, but their effect size was low. While we found above that both variables strongly affected small morphospecies, their effect on species composition was subtler than that of succession and sampling week.

## Discussion

There has been a rise in studies testing the interacting effects of dispersal and environmental change on diversity^46,47^, notably the importance of dispersal as a buffer against temperature warming^48,49^. However, the effect of temperature on the predicted hump-shaped diversity-dispersal relationship has remained an open, yet, timely question^15^. Based on Kneitel and Miller’s work^8^, we expected a flattening of the diversity-dispersal relationship with an increase in temperature. We did not observe such a flattening. Instead, diversity decreased steadily with temperature, independently of dispersal rate (Fig. 1). A sensible explanation is that increased competition also induces increased extinction rate, which lowered not only alpha-diversity, but also gamma-diversity, as is observed in our study. In turn, niche space will become available in the modified communities, creating a relaxation of competition that may maintain the hump-shaped pattern. Another unexpected result is the observation that temperature favored large-bodied species, which indicates that the temperature-size rule^33^ may not be the norm.

Among the recent contributions testing the link between dispersal rate and metacommunity diversity, only 6 studies^8,50–54^ were successful in capturing the hump-shaped relationship theoretically proposed by Mouquet and Loreau^7^. The main similarity between these six studies was that the initial diversity of each patch in the metacommunity was heterogeneous^55^. In our study, species diversity was homogenized before the start of the experiment, which can be visualized in Fig. 3 (notice the small size of both ellipses for the first week). We were still able to observe a hump-shaped relationship, except in a few treatments. These discrepant results suggest that the diversity-dispersal relationship may be context-dependent, and studies have shown that specific conditions influenced the presence of the hump-shaped relationship^50,53,56^. For instance, Vanschoenwinkel *et al*.^56^ observed this relationship only for passive-, but not active dispersers, and only if they were in highly disturbed environments. Note finally that the unimodal pattern in our experiment is subtler than predicted theoretically^7^. This may be due to the homogeneity in species composition and in environmental conditions at the start of the experiment, which differ from model assumptions^7^.

Since we used the same experimental design for the starting conditions in our early- and late- communities, it leads us to question why the early-successional community did not show a hump-shaped relationship in the low temperature treatments. Based on a mechanistic model, Haegeman and Loreau^9^ found two conditions where diversity will steadily increase with higher consumer dispersal, as with our early-successional communities in local temperature: (1) if consumer dispersal rate varies but resource dispersal rate remains constant, or (2) if niche overlap between consumer species is low, resulting in high levels of coexistence (this case is observed for the ‘spatial insurance hypothesis’ model of Loreau *et al*.^57^). We can exclude the first explanation as dispersal rates were the same for the consumers (protists) and resources (bacteria) in our experiment. However, the second explanation is plausible, as interspecific competition is expected to be less acute with lower temperature, and since indeed less extinctions were observed in these conditions.

We found a negative effect of temperature on evenness. This indicates that some species where positively selected by temperature and reached higher densities in greater temperatures. Interestingly, the body-size analyses showed that temperature favored larger morphospecies (see Electronic Supplementary Material, Figs. S6 and S7). This result is in disagreement with classical theory on temperature-size rule, i.e. that higher temperature benefits smaller organisms (see^33^), and may provide another exception to this relationship^58^. In the case of our protozoan-based system, it is plausible that a positive allometric scaling of attack rate with body size may disproportionately favor larger individuals with increased temperature, providing a competitive advantage for the large morphospecies.

When considering morphospecies composition, succession and sampling week had expectedly the strongest impact, with community structure being very different at the start of the experiment in early- and late- stages, and converging in similarity with time (Fig. 2). Contrary to our expectations, dispersal rate had a minor effect on composition, with little evidence of homogenization. Temperature had a stronger effect, which reflects its effect on small-bodied species. Interestingly, composition variability was highest in the intermediate sampling weeks, which is in agreement with the result on beta-diversity that was also highest during this period. The low beta-diversity at the start of the experiment is explained by the experimental design, as the communities in each tube and successional stage originated from the same species pool. The decrease in beta-diversity at the end of the experiment is likely the result of composition convergence, with a selection of the larger-bodied species. This observed temporal pattern of beta-diversity, as well as the decrease of alpha-diversity during the first weeks of the experiment (see Supplementary Information, Fig. S3) can be attributed to patch-dynamic mechanisms^30^. This element of the metacommunity theory is the paradigm that best matches our experiment. First, our experimental design used local environments that were similar, which largely prevents species sorting and mass effect from operating. Second, our chosen experimental dispersal rate was high enough to allow species to overcome dispersal limitation, but was too low for rescue- or mass- effects to influence local population dynamics. Third, the fact that community structure exhibited a clear temporal pattern, with trait-dependent extinctions, places our setting outside a neutral context^30^.

Our experiment is useful for understanding how biodiversity is organized in fragmented environments under temperature change. First, while we found the shape of the diversity-dispersal relationship to be variable, as shown in the theoretical work of Haegeman and Loreau^9^, our results largely corroborate the importance of intermediate levels of dispersal as an insurance for local diversity. This result highlights the importance of corridors in management schemes. Second, in the context of metacommunities, temperature increase may not affect local diversity through a disruption of the diversity-dispersal relationship, but merely by increasing rates of extinctions at both local- and global- scales. Small species, in particular, may be especially prone to temperature-induced extinction. Extending current theories^7,16^ to account for these effects will be useful for developing optimal conservation programs.

## Supplementary information

Supplementary Information for The effects of temperature and dispersal on species diversity in natural microbial metacommunities

## Data Availability

Data will be available in Dryad.
