# Supplementary material for: The effects of temperature and dispersal on species diversity in natural microbial metacommunities
Source: Sci Rep. 2019 Dec 4;9:18286. doi: 10.1038/s41598-019-54866-9 (PMC6892927; doi:10.1038/s41598-019-54866-9)
Supplement: Supplementary file 1 — Supplementary Information for The effects of temperature and dispersal on species diversity in natural microbial metacommunities [file 41598_2019_54866_MOESM1_ESM.pdf]

ELECTRONIC SUPPLEMENTARY MATERIAL FOR:

The effects of temperature and dispersal on species diversity in natural microbial metacommunities

Elodie C Parain; Sarah M Gray; Louis-Félix Bersier

Fig. S1 Temperature and Light settings in the four incubators 2

Fig. S2 Relationship between alpha- and gamma-diversity in the four dispersal levels 3

Table S1 List of protozoan morphospecies 4

Fig. S3 Gamma-, alpha-diversity, and total species richness as a function of sampling time 5

Table S2 Results of linear mixed-effects models for alpha-diversity as a function of successional stage, temperature, and the linear and quadratic terms of dispersal 6

Table S3 Results of linear mixed-effects models evenness and total density as a function of dispersal rate and temperature in each successional stage 7

Table S4 Results of generalized least squares models for gamma- and beta- diversity as a function of dispersal rate and temperature in each successional stage 8

Fig. S4 Evenness, total density, gamma- and beta- diversity as a function of dispersal rate for the four temperature levels 9

Table S5 Results of a Generalized Linear Mixed-Effects model for the proportion of extinctions as a function of body size of the morphospecies 11

Fig. S5 Proportion of extinctions in the three body-size categories 12

Table S6 Results of Generalized Linear Mixed-Effects models for the change in proportions of small vs. medium and large morphospecies with sampling weeks, dispersal, and temperature 13

Fig. S6 Change through time of the relative number of species in the two categories of body size according to dispersal and temperature in the early successional stage 14

Fig. S7 Change through time of the relative number of species in the two categories of body size according to dispersal and temperature in the late successional stage 15

Table S7 Results of the Multivariate Analysis of Variance on distance matrices for species composition 16

Fig. S1 Temperature and Light settings in the four incubators.

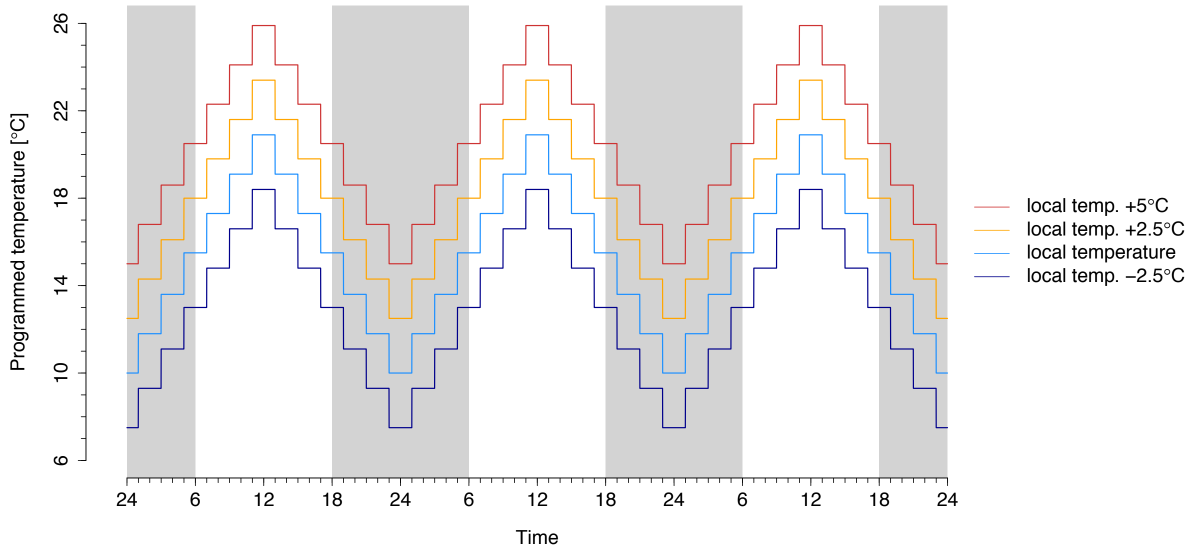

Temperature and light settings. The conditions are given for a sequence of three days, with the colored lines corresponding to the four temperature treatments. For the Local condition treatment, these temperature values were based on the natural June temperature of Champ Buet according to Worldclim (minimum: 10°C, average: 15.5°C, maximum: 20.9°C). For the remaining three incubators, the temperature values were adjusted according to treatment (-2.5°C, +2.5°C, or +5°C). Note that the temperatures used in the experiment were not lethal for the protists, with the highest temperature only being 25.9°C. To standardize day and night, all incubators had a 12 hour light:dark cycle (given by the shaded background).

Fig. S2 Relationship between alpha- and gamma-diversity in the four dispersal levels

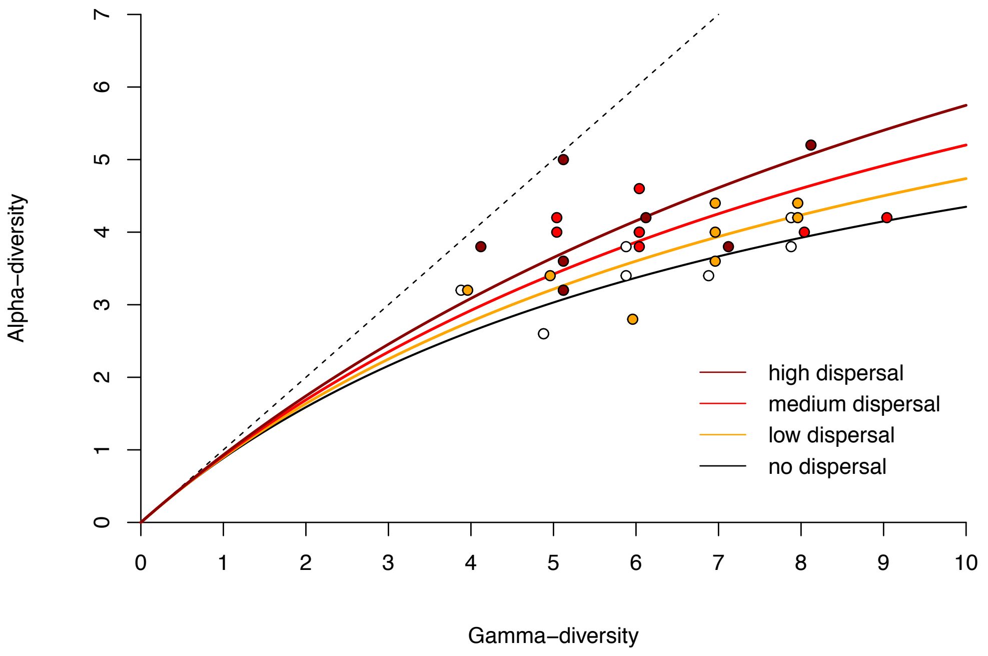

Relationship between alpha- and gamma-diversity as a function of dispersal treatment in the seventh week of the experiment. The lines are fitted one-parameter Michaelis-Menten equations [$Y=aX/(a+X)$, with Y the alpha-diversity and X the gamma-diversity], in this way preventing alpha-diversity to be larger than gamma-diversity. From no- to high- dispersal, the estimates and standard errors (in parentheses) of the parameter *a* are: 7.70 (0.57), 9.00 (0.73), 10.84 (1.71), 13.52 (2.96). For legibility, points for gamma-diversity are slightly shifted horizontally.

Table S1 List of morphospecies sampled in *Sarracenia purpurea* and used in the experiment.

Msp. Nr	morphospecies name	size	taxonomic category	early	late
msp01	Flagellata sp. 1	small	Flagellata	1	0
msp02	*Chrysomonadida* sp. 1	small	Flagellata	1	1
msp03	Flagellata sp. 2	small	Flagellata	1	1
msp04	Flagellata sp. 3	medium	Flagellata	1	0
msp05	*Colpidium* sp.	large	Ciliophora	1	1
msp06	*Bodo* sp.	small	Flagellata	1	1
msp07	Flagellata sp. 4	medium	Flagellata	1	0
msp08	*Euglena* cf. *gracilis*	large	Flagellata	1	1
msp09	*Colpoda* sp.	large	Ciliophora	1	1
msp10	Ciliophora sp. 1	large	Ciliophora	1	1
msp11	Flagellata sp. 5	small	Flagellata	1	1
msp12	Flagellata sp. 6	small	Flagellata	1	1
msp13	*Chrysomonadida* sp. 2	small	Flagellata	0	1
msp14	Flagellata sp. 7	small	Flagellata	0	1
msp15	Ciliophora sp. 2	large	Ciliophora	1	1
msp16	Bdelloid rotifer	large	Rotifera	1	1
msp17	Flagellata sp. 8	medium	Flagellata	1	1
msp18	Ciliophora sp. 3	large	Ciliophora	1	1

Legend: Msp. Nr: morphospecies number; size class: small (<15µm), medium (15µm-40µm), large (40µm-200µm); early and late: presence/absence in early- and late-successional communities, respectively.

Fig. S3 Average per tube ("alpha diversity"; black circles) and per treatment ("gamma diversity"; grey circles) and total species richness (white circles) as a function of sampling week in early- (left panel) and late-successional (right panel) communities.

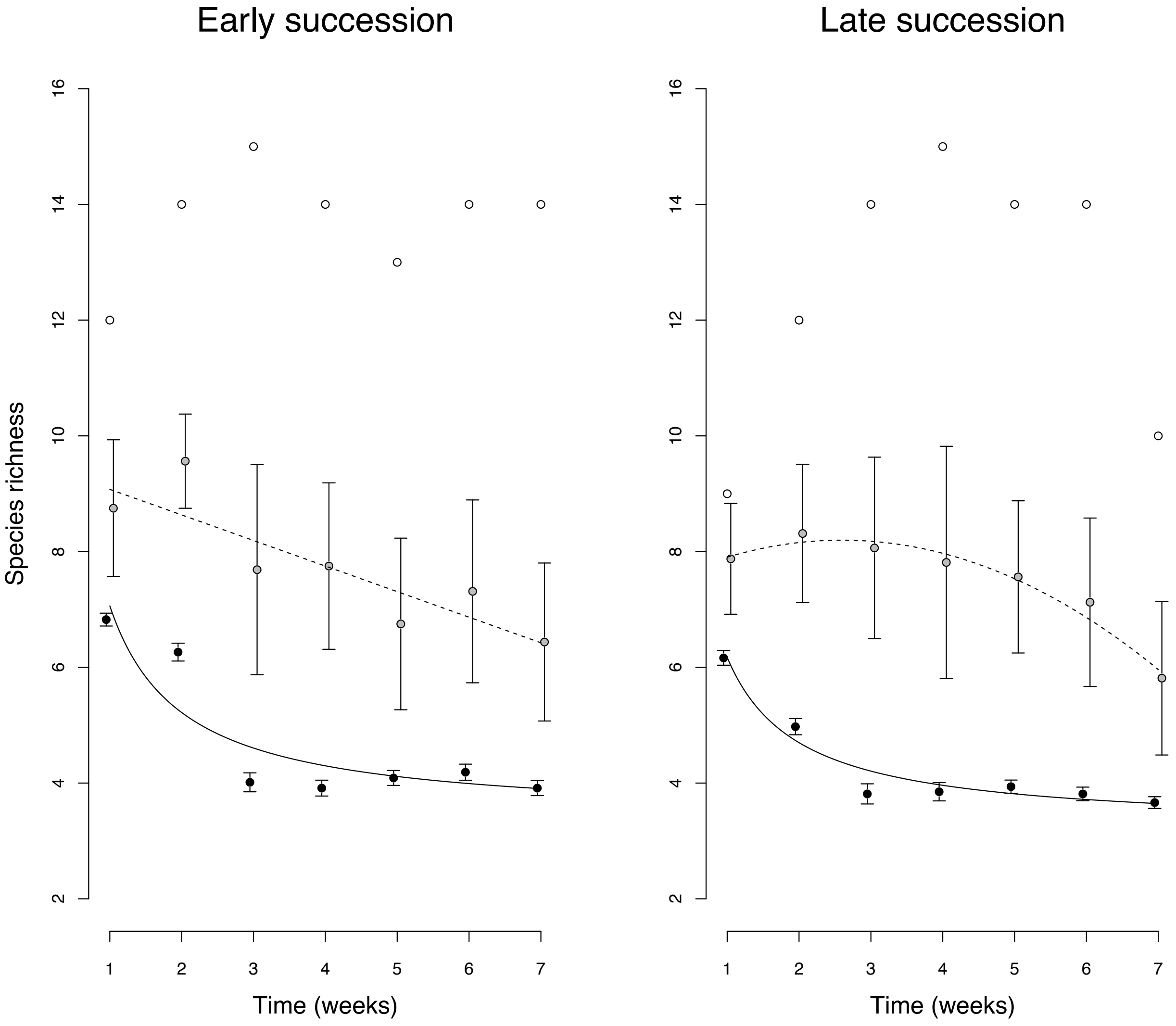

Error bars indicate ± one standard error. Total species richness is the cumulative number of species over all treatments in a given week. In both early- and late-successional stages, alpha-diversity decreased markedly with time, which can be captured by a hyperbolic relationship (fitted solid lines). Gamma-diversity (total number of species in the 5 tubes forming a metacommunity) also showed a negative trend, but with weaker decrease during the first sampling weeks (dashed lines describe a linear and a quadratic relationship for early- and late-successional stages, respectively). There was no evidence of a decrease in total species richness with time in both successional stages; the variability was attributable to rare species being undetected.

Table S2 Results of linear mixed-effects models for alpha-diversity as a function of successional stage, temperature, and the linear and quadratic terms of dispersal.

parameter	estimate	SE	t-value	p-value
intercept	3.94	0.19	21.19	<0.001
succession(late)	-0.34	0.18	-1.91	0.072
temperature	-0.41	0.10	-4.08	<0.001
dispersal	0.54	0.44	1.21	0.24
$(\text{dispersal})^2$	-0.52	0.39	-1.35	0.19
succession(late) : temperature	0.27	0.14	1.92	0.071
succession(late) : dispersal	-0.52	0.63	-0.83	0.42
succession(late) : $(\text{dispersal})^2$	0.87	0.55	1.60	0.13
temperature : dispersal	0.52	0.16	3.26	0.004
temperature : $(\text{dispersal})^2$	-0.14	0.051	-2.67	0.016
dispersal : $(\text{dispersal})^2$	0.13	0.085	1.56	0.14
succession(late) : temperature : dispersal	-0.51	0.23	-2.27	0.036
succession(late) : temperature : $(\text{dispersal})^2$	0.12	0.072	1.65	0.12
succession(late) : dispersal : $(\text{dispersal})^2$	-0.25	0.12	-2.06	0.054
$(\text{week})^{-1}$	3.18	0.14	23.20	<0.001
log(total density)	-0.17	0.044	-3.88	<0.001

Given is the best model after model selection based on AIC, starting with a model containing all interactions between the treatment variables (succession, temperature, and the linear and quadratic terms of dispersal). Analyses were performed at the tube level. Sampling week and total density were included as covariates. Sampling week was considered as a continuous variable and, to account for the sharp decrease in alpha diversity with sampling weeks (see Fig. S3), was reciprocally transformed (week^{-1}). To account for repeated measures, we modelled correlation between observations with an AR1 approach.

Table S3 Results of linear mixed-effects models for evenness and total density as a function of dispersal rate and temperature, separated for early- and late- successional communities.

response variable / successional stage	parameter	estimate	SE	t-value	p-value
evenness (log transformed)					
early	intercept	0.25	0.014	17.55	<0.001
	temperature	-0.016	0.0065	-2.49	0.028
	dispersal	0.0005	0.0043	0.11	0.92
	temp : disp	0.0053	0.0035	1.51	0.16
	$(\text{week})^{-1}$	0.19	0.013	15.43	<0.001
	log(total density)	-0.015	0.0037	-4.19	<0.001
late	intercept	0.15	0.017	9.03	<0.001
	temperature	-0.018	0.0038	-4.69	<0.001
	dispersal	0.042	0.013	3.20	0.008
	$(\text{dispersal})^2$	-0.014	0.0042	-3.38	0.006
	$(\text{week})^{-1}$	0.23	0.012	19.23	<0.001
	log(total density)	-0.02	0.0043	-3.95	<0.001
total density (log transformed)					
early	intercept	2.15	0.087	24.75	<0.001
	week	0.14	0.019	7.47	<0.001
late	intercept	2.62	0.14	19.23	<0.001
	temperature	0.25	0.095	2.61	0.026
	dispersal	0.027	0.19	1.44	0.18
	$(\text{dispersal})^2$	-0.089	0.060	-1.49	0.17
	temp : disp	-0.26	0.15	-1.67	0.13
	temp : $(\text{disp})^2$	0.079	0.049	1.61	0.14
	week	0.097	0.018	5.47	<0.001

Given is the best model after model selection based on AIC, starting with a model with all interactions between temperature and the linear and quadratic terms of dispersal. Both response variables were log transformed to satisfy normality assumption; this assumption was not reached for total density in the late succession and the p-values must be interpreted with caution. Sampling week (reciprocally transformed for evenness) and total density were included as covariates. Repeated measures were modelled with an AR1 correlation approach between observations.

Table S4 Results of generalized least squares models for gamma- and beta-diversity as a function of dispersal rate and temperature, separated for early- and late- successional communities.

response variable / successional stage	parameter	estimate	SE	t-value	p-value
gamma diversity					
early	intercept	12.10	0.90	13.41	<0.001
	temperature	-0.39	0.25	-1.55	0.12
	dispersal	0.066	0.17	0.40	0.69
	temp : disp	0.20	0.14	1.46	0.15
	week	-0.34	0.080	-4.24	<0.001
	log(total density)	-0.68	0.20	-3.43	0.001
late	intercept	7.39	0.59	12.61	<0.001
	temperature	-0.052	0.23	-0.22	0.85
	dispersal	0.12	0.15	0.78	0.48
	temp : disp	-0.24	0.12	-1.97	0.070
	week	0.64	0.31	2.09	0.039
	$(\text{week})^2$	-0.12	0.037	-3.22	0.002
beta diversity (Box-Cox transformed)					
early	intercept	-0.81	0.22	-3.67	<0.001
	week	0.51	0.076	6.65	<0.001
	$(\text{week})^2$	-0.054	0.0092	-5.84	<0.001
	log(total density)	-0.11	0.046	-2.34	0.021
late	intercept	-1.32	0.14	-9.53	<0.001
	temperature	0.050	0.052	0.96	0.34
	dispersal	-0.008	0.034	-0.24	0.81
	temp : disp	-0.48	0.028	-1.75	0.083
	week	0.65	0.073	8.94	<0.001
	$(\text{week})^2$	-0.075	0.009	-8.50	<0.001

Both diversity indices were measured at the treatment level (4 temperature x 4 dispersal) at each sampling week. Beta-diversity was Box-Cox transformed. Sampling week and total density were included as covariates. Sampling week was considered a continuous variable, with a quadratic term necessary for beta- and gamma-diversity in the late-successional stage (see Fig. S3). To account for repeated measures, we modelled correlation between observations with an AR1 approach.

Fig. S4 Evenness, total density, gamma-diversity, and beta-diversity as a function of dispersal rate for the four temperature levels and in the two successional stages.

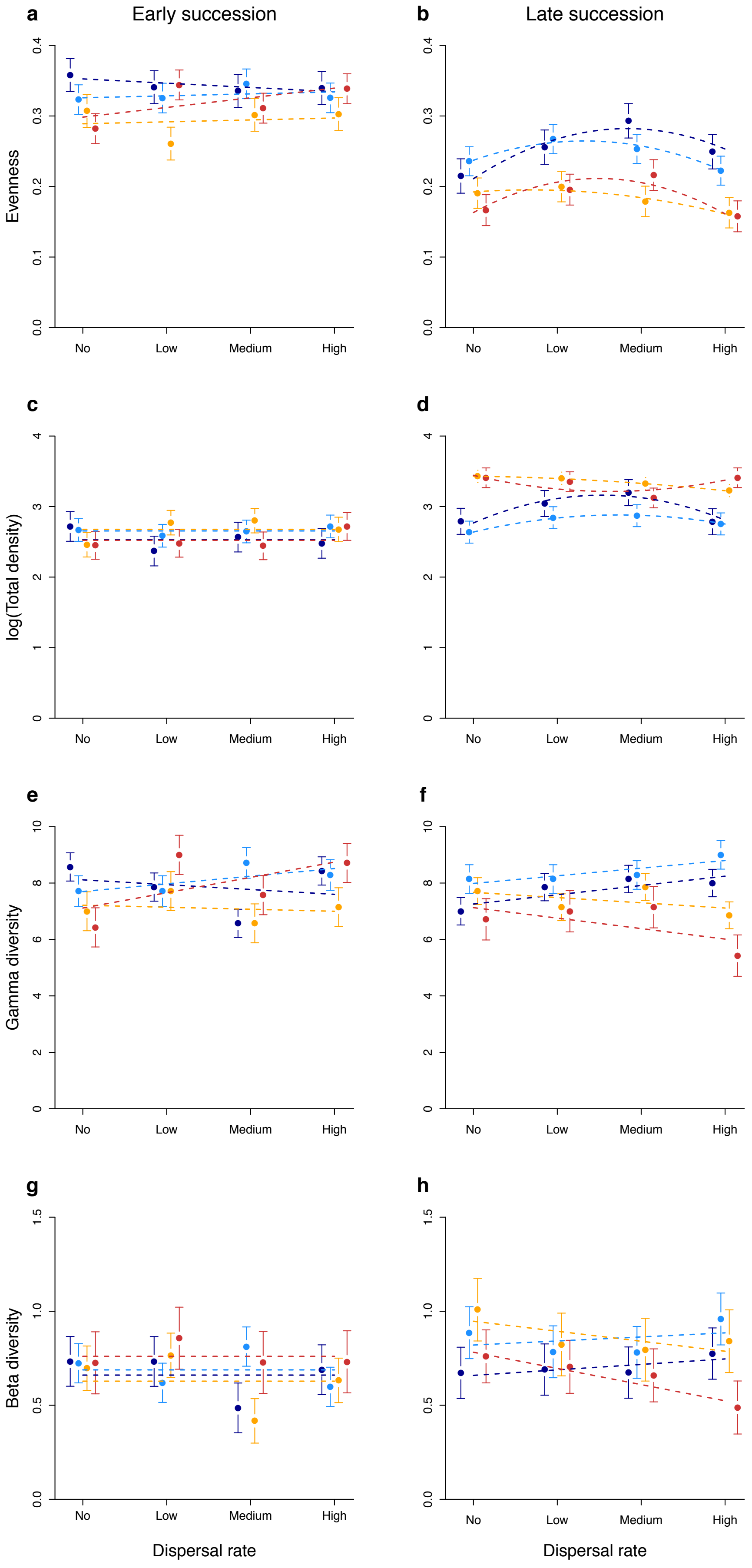

Legend of Fig. S4.

Relationships between (**a**, **b**) Evenness, (**c**, **d**) log-transformed total density, (**e**, **f**) gamma-diversity, and (**g**, **h**) beta-diversity and dispersal rate for the four temperature levels and in the two successional stages. The temperature levels are represented as Local Temperature (light blue), Local Temperature - 2.5 °C (dark blue), Local Temperature + 2.5°C (orange), Local Temperature + 5°C (red). Error bars indicate one standard error. The dashed lines are results of quadratic regressions (**b**, **d**), linear regression (**a**, **e**, **f**, **h**), and mean value (**c**, **g**), according to the statistical results in Tables S3 (**a** to **d**) and S4 (**e** to **f**).

Table S5 Results of a Generalized Linear Mixed-Effects model (binomial family) for the proportion of extinctions as a function of morphospecies body size.

parameter	estimate	SE	z-value	p-value
intercept	1.45	2.34	0.61	0.53
size (linear)	-5.05	1.39	-3.62	<0.001
size (quadratic)	-2.13	0.81	-2.63	0.009

Taxonomic status (see Table S1) was used as a random factor (the Bdelloid rotifer is excluded from the analysis). Size was entered as an ordered variable (ordered by increasing body size: small to medium to large). The significant linear term indicates that small morphospecies experienced higher extinction rates. A logistic regression performed exclusively for the Flagellata morphospecies confirms this result (for this analysis, size was considered a quantitative variable with category small=1, medium=2, and large=3). Fig. S5 represents the proportions of extinction in the three size categories.

Fig. S5 Proportion of extinctions in the three body-size categories.

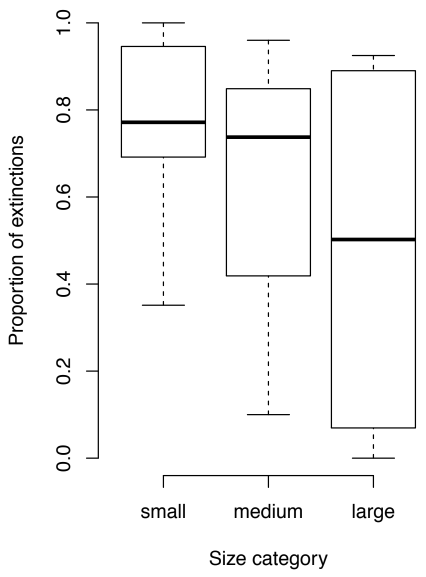

Boxes give minimum, 1st quartile, median, 3rd quartile, and maximum values; if observations fall outside 1.5 times the interquartile range, they are displayed as dots.

Table S6 Results of Generalized Linear Mixed-Effects models (binomial family) for the change in proportions of small vs. medium and large morphospecies with sampling week, temperature, and dispersal, in early and late communities.

successional stage	parameter	estimate	SE	z-value	p-value
early	intercept	-0.70	0.038	-18.45	<0.001
	dispersal	-0.042	0.13	-0.33	0.74
	$(\text{dispersal})^2$	-0.088	0.12	-0.71	0.48
	temperature	-0.13	0.038	-3.52	<0.001
	week	-0.15	0.036	-4.21	<0.001
	disp : temp	-0.37	0.13	-2.96	0.003
	$(\text{disp})^2$: temp	0.31	0.12	2.51	0.012
	temp : week	-0.11	0.036	-3.10	0.002
late	intercept	-1.10	0.22	-5.10	<0.001
	dispersal	0.64	0.54	1.19	0.23
	$(\text{dispersal})^2$	-0.93	0.61	-1.53	0.13
	temperature	-0.43	0.048	-8.88	<0.001
	week	-0.58	0.044	-13.27	<0.001
	disp : $(\text{disp})^2$	0.093	0.22	0.42	0.67
	disp : temp	0.17	0.14	1.22	0.22
	disp : week	-0.43	0.15	-2.87	0.004
	$(\text{disp})^2$: temp	0.45	0.14	3.15	0.002
	$(\text{disp})^2$: week	-0.57	0.15	-3.86	<0.001
	temp : week	-0.21	0.043	-4.90	<0.001
	$(\text{disp})^2$: temp : week	-0.16	0.048	-3.27	0.001

Given are the best models after model selection based on AIC, starting with a model with all interactions between sampling week (week), temperature (temp) and the linear and quadratic terms of dispersal (disp and $(\text{disp})^2$, respectively). Explanatory variables were standardized. The response variable was the number of small- vs. the number of medium- plus large-sized morphospecies in each tube at each sampling week. Medium- and large-sized species were pooled as their response to the treatments were qualitatively similar (see Fig. 2 in the main text). To account for repeated measures, we used tube identity as a random factor. The results for the change in proportions with sampling weeks as a function of temperature and dispersal in early- and late- successions are shown in Fig. S6 and S7.

Fig. S6 Change through time (sampling week) of the proportions of morphospecies in the two categories of body size (small vs. medium and large morphospecies) according to (**a**) temperature and (**b**) dispersal for the **early-successional** stage.

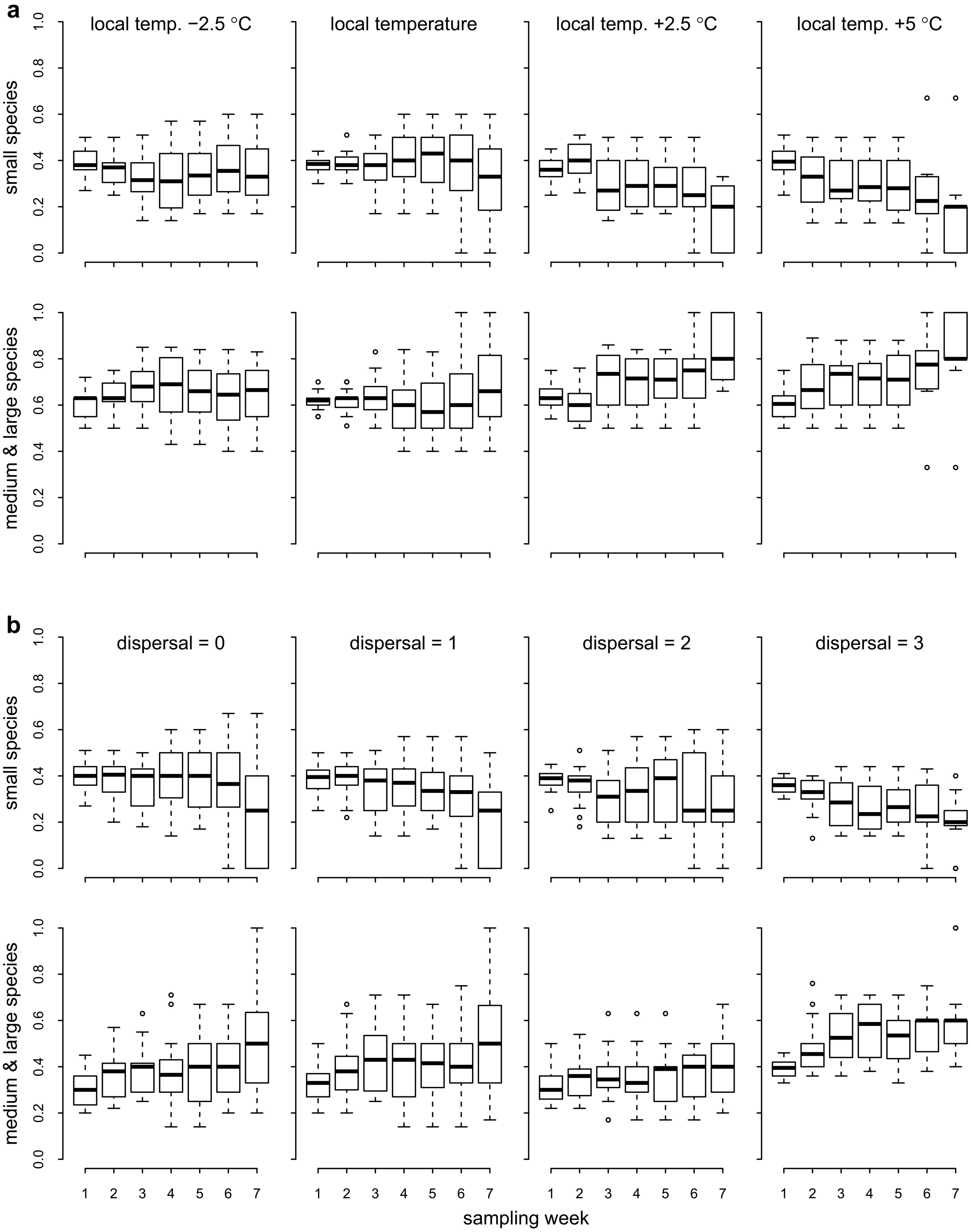

Legend: 0 = no dispersal; 1 = low dispersal; 2 = medium dispersal; 3 = high dispersal. Proportion of species is expressed as percentages within each tube. Boxes as in Fig. S5.

Fig. S7 Change through time (sampling week) of the proportions of species in the two categories of body size (small vs. medium and large morphospecies) according to (**a**) temperature and (**b**) dispersal for the **late-successional** stage.

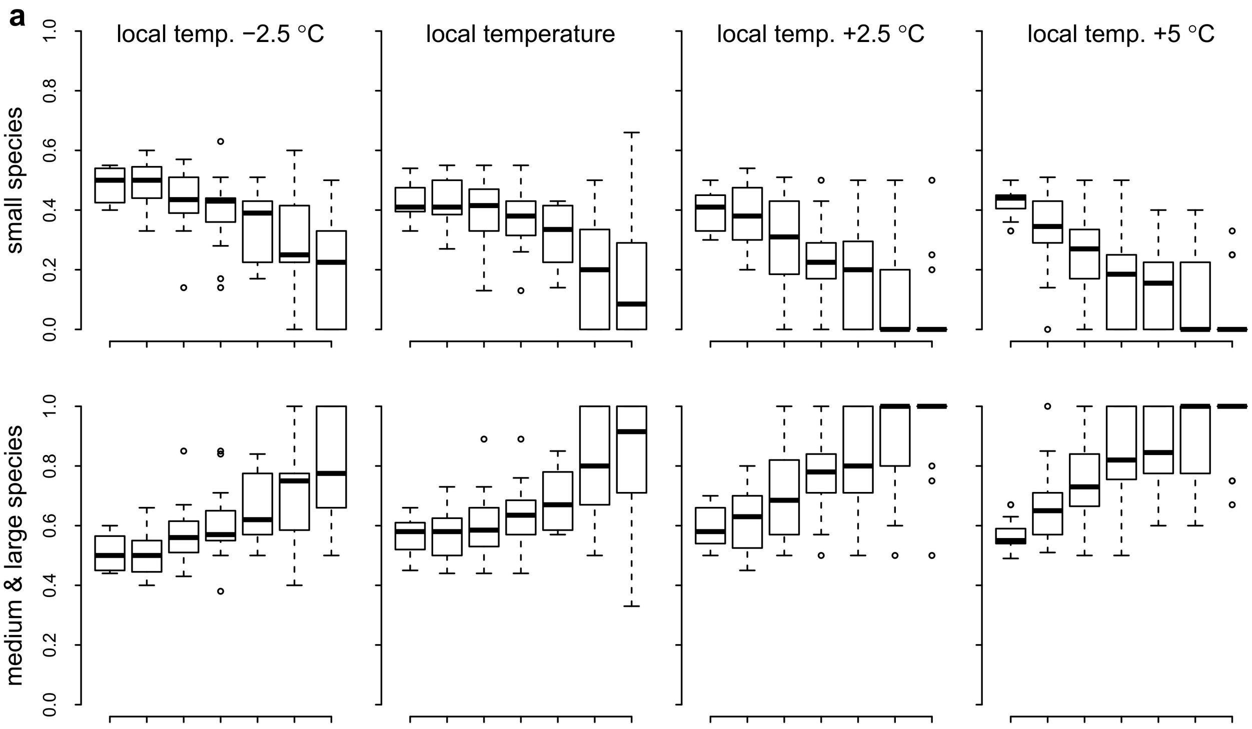

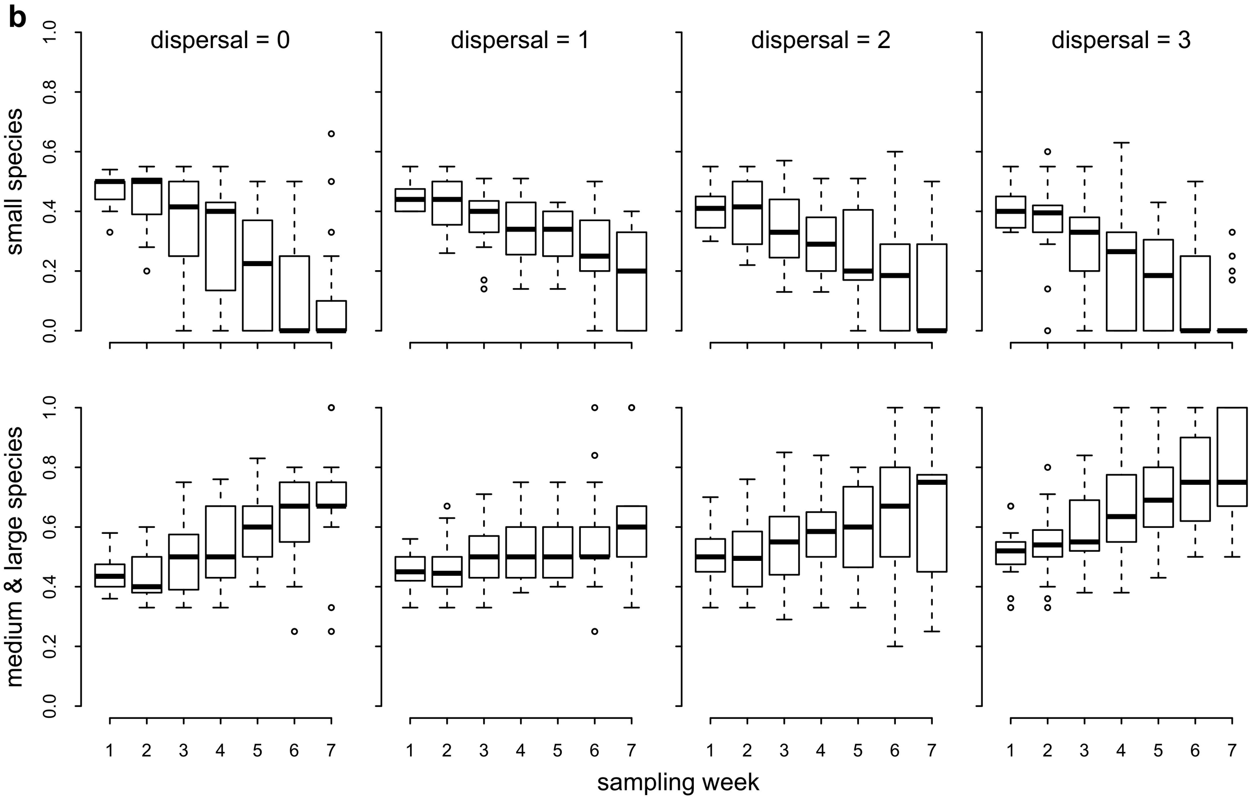

Legend: 0 = no dispersal; 1 = low dispersal; 2 = medium dispersal; 3 = high dispersal. Proportion of species is expressed as percentages within each tube. Boxes as in Fig. S5.

Table S7 Results of the "adonis" Multivariate Analysis of Variance on distance matrices for species composition.

parameter	df	SS	MS	F	R^2	p-value
succ	1	33.74	33.74	266.82	0.12	<0.001
temp	3	8.09	2.70	21.34	0.03	<0.001
disp	3	2.94	0.98	7.74	0.01	<0.001
week	6	55.50	9.26	73.21	0.20	<0.001
succ : temp	3	3.31	1.10	8.73	0.01	<0.001
succ : disp	3	2.00	0.67	5.27	0.01	<0.001
succ : week	6	20.20	3.37	26.61	0.07	<0.001
temp : disp	9	3.11	0.35	2.74	0.01	<0.001
temp : week	18	7.83	0.44	3.44	0.03	<0.001
disp : week	18	2.72	0.15	1.20	0.01	0.095
residuals	1049	132.65	0.13		0.49	
total	1119	272.13			1.00	

Analysis was performed for the whole dataset (160 tubes measured over 7 weeks). Legend: succ, successional stage; temp, temperature treatment; disp, dispersal treatment; week, sampling week; the colon sign indicates the interaction between the terms. Only pairwise interactions between the explanatory variables were considered. The results highlight the major effects on species composition of succession, sampling week, and of their interaction, which can be visualized in the Fig. 3 in the main text. Note that highly significant p-values are often achieved with very small percentages of explained variance (R^2) due to the large sample size.
